# Rib fractures in the elderly population: a systematic review

**DOI:** 10.1007/s00402-022-04362-z

**Published:** 2022-02-08

**Authors:** Ruben J. Hoepelman, Frank J. P. Beeres, Marilyn Heng, Matthias Knobe, Björn-Christian Link, Fabrizio Minervini, Reto Babst, Roderick. M. Houwert, Bryan J. M. van de Wall

**Affiliations:** 1 Department of Trauma Surgery, University Medical Center Utrecht, Utrecht, the Netherlands; 2 Department of Orthopedics and Trauma Surgery, Luzerner Kantonsspital, Spitalstrasse 16, 6000 Lucerne, Switzerland; 3 Department of Health Sciences and Medicine, University of Lucerne, Lucerne, Switzerland; 4 Department of Orthopedic Surgery, Orthopedic Trauma Initiative, Harvard Medical School, Massachusetts General Hospital, Boston, MA USA

**Keywords:** Rib fractures, Rib fixation, Conservative treatment, Systematic review

## Abstract

**Background:**

Multiple rib fractures are associated with significant morbidity and mortality, especially in elderly patients. There is growing interest in surgical stabilization in this subgroup of patients. This systematic review compares conservative treatment to surgical fixation in elderly patients (older than 60 years) with multiple rib fractures. The primary outcome is mortality. Secondary outcomes include hospital and intensive care length of stay (HLOS and ILOS), duration of mechanical ventilation (DMV) and pneumonia rates.

**Methods:**

Multiple databases were searched for comparative studies reporting on conservative versus operative treatment for rib fractures in patients older than 60 years. Both observational studies and randomised clinical trials were considered.

**Results:**

Five observational studies (*n* = 2583) were included. Mortality was lower in operatively treated patients compared to conservative treatment (4% vs. 8%). Pneumonia rate and DMV were similar (5/6% and 5.8/6.5 days) for either treatment modality. Overall ILOS and HLOS of stay were longer in operatively treated patients (6.5 ILOS and 12.7 HLOS vs. 2.7 ILOS and 6.5 ILOS). There were only minimal reports on perioperative complications. Notably, the median number of rib fractures (8.4 vs. 5) and the percentage of flail chest were higher in operatively treated patients (47% vs. 39%).

**Conclusion:**

It remains unknown to what extent conservative and operative treatment contribute individually to reducing morbidity and mortality in the elderly with multiple rib fractures. To date, the quality of evidence is rather low, thus well-performed comparative observational studies or randomised controlled trials considering all confounders are needed to determine whether operative treatment can improve a patient’s outcome.

**Supplementary Information:**

The online version contains supplementary material available at 10.1007/s00402-022-04362-z.

## Introduction

Rib fractures are a common entity among patients with thoracic injury. Due to the optimisation of trauma care in the last decade, overall mortality rates have dropped significantly and currently lay around 5% to 7% in the United States and Netherlands [1–3].

Morbidity and mortality rates, however, remain considerably high among elderly patients [2, 3]. Due to an aging population, it is expected that this will become a larger problem, thus demanding further optimisation of care in this frail subgroup of patients [4]. Conservative treatment consisting of (non)invasive ventilation, pain management and physiotherapy, has long been considered the gold standard for the management of rib fractures [4, 5]. Since the development of multiple plating and rib fixation systems, there has been a growing interest in operative treatment, also for elderly patients with rib fractures [6, 7]. General indications for operative treatment of rib fractures include, flail chest segment, reduction of pain and disability, chest wall deformity, thoracotomy and open rib fractures [8]. However, it is a controversial topic and it remains to be seen whether these patients benefit from surgical fixation.

A recent meta-analysis reported promising results after operative treatment in regards to reducing mortality, pneumonia rate, Duration of mechanical ventilation (DMV) and Intensive care length of stay (ILOS), however, did not specifically analyze outcomes for elderly patients [9]. Clinical studies comparing operative treatment to conservative treatment in the elderly are scarce. The few clinical studies that have been published are relatively small and lack the power for drawing solid conclusions. For this reason, we performed this systematic review.

The aim of this systematic review is to compare surgical rib fixation to conservative treatment in patients 60 years and older with multiple rib fractures. The primary outcome is the mortality rate. Secondary outcomes are pneumonia rates, Hospital length of stay (HLOS), ILOS, DMV and perioperative complications.

## Methods

This systematic review was written according to the Preferred Reporting Items for Systematic Reviews and Meta-analysis (PRISMA) checklist [10]. No ethical approval was required for this systematic review.

### Search strategy and selection criteria

The PubMed/Medline, Embase, CENTRAL, and CINAHL databases were searched on 25 May 2021, for comparative studies reporting on conservative versus operative treatment for rib fractures in the elderly. The search syntax is provided in Online resources Table 1. Two reviewers (RJH, BJMvdW) screened the title and abstract for eligibility independently. Both randomised clinical trials and observational studies were considered for inclusion. Both reviewers independently performed full-text screening. Inclusion criteria were conservative versus operative treatment of two rib fractures or more in elderly patients (60 years or older), reporting on mortality rate and on secondary outcomes (pneumonia rate, other complications, HLOS, ILOS, DMV). The cutoff point of 60 was chosen to include a broader selection of studies. Exclusion criteria were languages other than English or Dutch, no availability of full-text, letters, meeting proceedings, and case series with fewer than ten patients. Disagreements on the eligibility of full-text articles were resolved by consensus or by a discussion with a third reviewer (FJB). Cross referencing of all included studies was performed to identify studies not found in the original search.

### Data extraction and quality assessment

Two reviewers (RJH, BJMvdW) independently performed data extraction. The following baseline characteristics were extracted from the included studies; first author, year of publication, study design, number of included patients, gender, age, number of fractured ribs, flail chest percentage, Injury severity score (ISS) and Abbreviated injury scale (AIS). In cases of studies that reported on the study population of interest as part of a subgroup analysis, only data from that particular analysis was used for the data synthesis of the present systematic review. Two reviewers (RJH, BJMvdW) independently assessed the methodological quality of included studies using the Methodological Index for Non-Randomised Studies (MINORS) [11]. Disagreements were resolved by consensus. Details on methodological quality assessment are provided in Online resources Table 2.

### Outcomes measures

The primary outcome was the mortality rate. Secondary outcomes include pneumonia rate, HLOS, ILOS, DMV and all other complications. None of the included studies specified/clearly defined pneumonia nor described other complications using uniform definitions. Therefore, rates were taken as reported.

### Statistical analysis

Information about continuous variables was presented as means with standard deviation (SD) or range, or information was converted to mean and SD using the methods described in the Cochrane Handbook for Systematic Reviews of Interventions [12]. Dichotomous variables were presented as counts and percentages. No p-values were calculated as this review was designed for explorative and descriptive purposes.

For all outcomes, the weighted mean or percentage was calculated according to the size of each study population. All outcome variables were ordered for methodological quality or alphabetically when equal and displayed in tables.

## Results

### Search

**Fig. 1:**
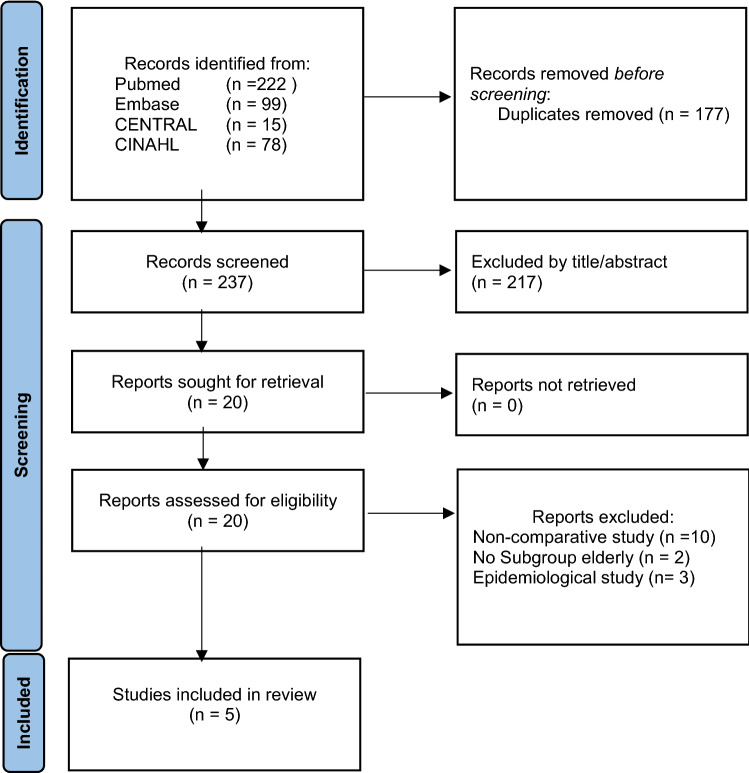
PRISMA flow diagram representing the search and screening process of articles comparing nonoperative to operative treatment in elderly. From Page et al. [10]

Figure 1 presents the flowchart of the literature search and study selection. A total of five articles were included. All articles were retrospective observational studies [13–17].

### Study characteristics

The five studies included 2583 patients; 1562 received conservative treatment and 1021 received operative treatment. Operatively treated patients were younger (71 vs. 74.9 years), had more males (68% vs. 63%) and had higher ISS scores (16.5 vs. 14.1). Notably, a median number of rib fractures (8.4 vs. 5) and the percentage of flail chest were higher in operatively treated patients (47% vs. 39%) (Table 1). Stratification for these characteristics was not possible as none of the studies reported outcomes separately for these specific subgroups.

**Table 1:** Studies on comparing conservative and operative treatment of rib fractures

Author	Year	Country	Number of patients	Male (%)	Age, IQR/SD	ISS, IQR/SD	Flail chest (%)	Fractured ribs, IQR/SD	Mortality (%)	Pneumonia (%)	HLOS days, IQR/SD	ILOS days, IQR/SD	DMV days IQR/SD
*Conservative treatment*													
Fitzgerald [14]	2017	USA	50	NR	75 (65–97)	19 (14–23)	NR	NR	2 (4)	7 (14)	17 (10–23)	12 (7–17)	NR
Chen Zhu [13]	2020	USA	758	518 (68)	72 (68–79)	NR	348 (46)	NR	55 (7.3)	8 (1)	7 (4–12)	4 (2–8)	7 (3–14)
Pieracci [15]	2021	USA	227	116 (51)	86 (80–99)	13 (4–34)	36 (16)	5 (1–17)	21 (9)	9 (4)	6	0	NR
Ali-Osman [17]	2018	USA	135	73 (54)	72 (66–81)	14 (8–24)	NR	5 (3–7.25)	13 (9.6)	16 (12)	4.8 (2.9–8.4)	4 (3–7)	4 (1–10)
Kane [16]	2018	USA	392	NR	75.4 ± 6.8	14.1 ± 10.3	NR	NR	33 (8)	54 (14)	5 (3–9)	0 (0–3)	NR
Weighted subtotal			1562	63%	74.9	14.1	39%	5	8%	6%	6.5 days	2.7 days	6.5 days
*Operative treatment*													
Fitzgerald [14]	2017	USA	23	NR	68 (63–89)	21 (16–26)	NR	NR	0 (0)	0 (0)	18 (14–23)	8 (5–11)	NR
Chen Zhu [13]	2020	USA	758	530 (70)	72 (68–78)	NR	345 (46)	NR	32 (4)	23 (3)	13 (9–18)	7 (4–13)	6 (2–13)
Pieracci [15]	2021	USA	133	81 (61)	84 (80–100)	14 (4–57)	76 (57)	9 (1–30)	10 (8)	16 (12)	11	4.5	NR
Ali-Osman [17]	2018	USA	64	41 (61)	69 (63–74)	17.5 (9–25)	NR	7 (5.25–9)	1 (2)	5 (8)	12 (9–16)	6 (3–10)	3 (1–15)
Kane [16]	2018	USA	43	NR	71.3 ± 6.0	20.1 ± 8.5	NR	NR	1 (2)	2 (5)	12 (10–16)	5 (0–8)	NR
Weighted subtotal			1021	68%	71	16.5	47%	8.4	4%	5%	12.7 days	6.5 days	5.8 days

*NR* not reported; *ISS* injury severity score; *HLOS* hospital length of stay; *ILOS* intensive care unit length of stay; *DMV* duration mechanical ventilation

The studies included multi-trauma patients and patients with isolated rib fractures. One study reported on chest AIS and non-chest AIS, patients with Non-chest AIS > 2 were excluded [13]. Another study did not report on AIS scores, however, did exclude patients with Head AIS > 1 or Chest AIS < 3 [15]. Two studies only included patients with three or more rib fractures and did not further mention AIS scores [16, 17]. Two studies reported stratified ISS scores [13, 17]. Details are available in Online resources Table 3.

### Quality assessment

The details and distribution of the MINORS scores are described in Online resources Table 4. The average MINORS score was 13 ± 1.3 (range 12–15).

## Outcomes

### Mortality

All five studies (*n* = 2583) reported on mortality. The overall weighted mean mortality rate in operatively treated patients was 4% versus 8% in the conservative group. (Table 1).

### Secondary outcomes

Pneumonia rate, HLOS and ILOS were reported in all five studies (*n* = 2583). The weighted pneumonia rate was 5% in operatively treated patients and 6% in conservatively treated patients. HLOS and ILOS were 12.7 and 6.5 days for operatively treated patients and 6.5 and 2.7 for conservatively treated patients.

Two studies reported on the duration of mechanical ventilation [13, 17]. Operatively treated patients had a DMV of 5.8 days compared to 6.5 days in conservatively treated patients.

### Other reported complications

One study reported 7 (3.5%) versus 6 (3%) cases of pleural effusion, 7 (3.5%) cases versus 1 (0.5%) case of atrial fibrillation for conservative and operative treatment respectively [17]. There was 1 (0.5%) case of abscess and one case (0.5%) of pneumothorax for operatively treated patients and none for conservatively treated patients. Finally, there were two cases of arrhythmia, one in each treatment modality.

One study reported 10 (1.3%) versus 13 (1.7%) cases of acute respiratory distress syndrome (ARDS), 6 (0.8%) versus 12 (1.6%) cases of the decubitus ulcer, 10 (1.3%) versus 13 (1.7%) cases of sepsis and sixteen (2.1%) versus 30 (4%) cases of venous thromboembolism (VTE) in conservatively and operatively treated patients respectively [13].

One study reported 7 (14%) cases of pleural effusion and 19 (38%) cases of recurrent pneumothorax versus zero for either complication in operatively treated patients [14]. No other complications were reported.

## Discussion

This systematic review of five comparative observational studies describes outcomes of conservative and operative treatment for rib fractures in elderly patients older than 60 years of age. Mortality was potentially lower in operatively treated patients (4% vs. 8%). However, HLOS and ILOS seem shorter in conservatively treated patients (6.5 vs. 12.7 days and 2.7 vs. 6.5 days). There were minimal differences in pneumonia rate and DMV (5% vs. 6% and 5.8 vs. 6.5 days) between both treatment modalities and there were only minimal reports on other perioperative complications. Results however should be interpreted taking critical differences in baseline characteristics, described in the following sections, into account.

### Comparison to previous literature

To our knowledge, there currently is no systematic review on rib fractures in elderly patients. A recent systematic review and meta-analysis were published for flail chest and multiple rib fractures [9]. Similar to our review, they reported reduced mortality (Risk ratio (RR) 0.41) in operatively treated patients. However, in contrast to our systematic review, they found a reduced pneumonia rate (RR 0.59) and shorter ILOS in the operative group. Since this study encompasses all adults and not just elderly, the results are only moderately suited for comparison.

### Interpretation of results

As described previously, the differences in outcomes between the two treatment modalities found in the present review should be viewed from a distinct perspective. Factors such as ISS score, flail chest, number of rib fractures are known to influence both treatment choice and development of morbidity and mortality. As these factors differed between both treatment groups in the present study, it is difficult to distillate how much each factor contributed to the reported outcomes and, as a result, the causal inference of conservative and operative treatment.

For example, ILOS and HLOS indeed were shorter among conservatively treated patients. It is likely that the lower ISS score, lower number of rib fractures and flail chest among conservatively treated patients played a vital role in the observed difference. We tried to stratify for the differences in baseline characteristics, however, this was not possible with the available data.

Interestingly, the inverse applies to mortality. Despite the high ISS score, number of rib fractures and flail chest among operatively treated patients, mortality was lower after operative treatment. Acute trauma and intensive care management have drastically improved in the last decades, which has resulted in an overall decline in mortality in trauma patients [1, 18, 19]. It might very well be possible that the reduced mortality rates found among operatively treated patients are a result of these developments. To what extent the operative rib fracture treatment itself contributes to this reduction remains unknown.

All in all, based on the results found in this study it is difficult to determine which treatment is superior. Since it remains unknown which patients benefit from operative treatment, especially in elderly patients, a multidisciplinary approach is advisable [20]. The clinical outcome of patients is dependent on multiple factors including the severity of trauma and concomitant injuries, number of rib fractures, type and timing of treatment and adequacy of polytrauma care [1–3, 5, 21]. To distillate the pure contribution of conservative and operative treatment in this multifactorial causal relation, all these factors should be taken into account in future studies and analyses.

### Limitations

As mentioned previously, important confounders (number of rib fractures and incidence of flail chest) were not balanced equally across treatment groups, and since the definition for flail chest differed between studies, or in some cases, was even absent, the pooling of data and performing a meta-analysis was not feasible. Furthermore, the overall methodological quality of included studies was poor (Online resources Table 4). There were no randomised clinical trials and the comparative observational studies that were included frequently lacked clear definitions of outcomes and correction for confounders. Finally, the results are applicable to the entire spectrum of elderly patients with rib fractures; they give an impression of what may be expected in general of both treatment regimes. Results cannot be used on an individual patient level as there is much to be investigated before hard statements can be made about whom to operate.

## Conclusion

It remains unknown to what extent conservative and operative treatment contribute individually in reducing morbidity and mortality in the elderly with multiple rib fractures. To date quality of evidence is rather low and well-performed comparative observational studies or randomised controlled trials taking into account all described confounders are needed to determine whether operative treatment can improve patient outcomes. The indication for surgical treatment of multiple rib fractures remains elusive in current trauma surgery.

## Supplementary Information

Below is the link to the electronic supplementary material.

Supplementary file1 (DOCX 17 KB)

Supplementary file2 (DOCX 16 KB)

Supplementary file3 (DOCX 18 KB)

Supplementary file4 (DOCX 17 KB)

## Data Availability

The data supporting the findings of this study are available within the article and its supplementary materials.
