# Supplementary material for: Rib fractures in the elderly population: a systematic review
Source: Arch Orthop Trauma Surg. 2022 Feb 8;143(2):887–93. doi: 10.1007/s00402-022-04362-z (PMC9925562; doi:10.1007/s00402-022-04362-z)
Supplement: Supplementary file 1 — Supplementary file1 (DOCX 17 KB) [file 402_2022_4362_MOESM1_ESM.docx]

**Online resources Table 1.** Syntax of literature search on rib fractures in elderly patients

| Database | Syntax |
| --- | --- |
| **Pubmed/MEDLINE (n=222)** | (((elderly[Title/Abstract]) OR (65[Title/Abstract] AND older[Title/Abstract])) OR (geriatric[Title/Abstract])) AND (((((fractur* [Title/Abstract]) AND ((ribs [Mesh]) )) OR ((((rib fractures [Mesh]) OR flail chest [Mesh]) OR rib fractur* [Title/Abstract]) OR "flail chest" [Title/Abstract])))) |
| **Embase (n=99)** | (elderly:ti,ab OR (65:ti,ab AND older:ti,ab) OR geriatric:ti,ab) AND (rib:ti,ab AND fracture:ti,ab OR flail chest:ti,ab) |
| **CINAHL (n=78)** | (AB (elderly OR ( 65 years and older ) OR geriatric) AND  (AB rib fracture OR AB flail chest) |
| **CENTRAL (n=15)** | (Elderly:ti,ab OR geriatric:ti,ab OR (65:ti,ab AND Older:ti,ab)) AND (Rib:ti,ab AND Fracture:ti,ab) OR (Flail:ti,ab AND Chest:ti,ab) |

# Rib fractures in the elderly population: A systematic review.

**Journal: Archives of Orthopaedic and Trauma Surgery**

Ruben J. Hoepelman^1,2^, Frank J.P. Beeres^2,3^, Marilyn Heng^4^, Matthias Knobe^2^, Björn-Christian Link^2^, Fabrizio Minervini^2^, Reto Babst^2,3^, Roderick. M. Houwert^1^, Bryan J.M. van de Wall ^2,3,^

1. Department of Trauma Surgery, University Medical Center Utrecht, Utrecht, the Netherlands

2. Department of Orthopedics and Trauma Surgery, Luzerner Kantonsspital, Lucerne, Switzerland

3. University of Lucerne, Department of Health Sciences and Medicine, Lucerne, Switzerland.

4. Department of Orthopedic Surgery, Harvard Medical School, Orthopedic Trauma Initiative, Massachusetts General Hospital, Boston, Massachusetts, USA

**Corresponding author:**

Bryan J.M. van de Wall, MD, PhD, E-mail address: Bryan.vandewall@luks.ch
