# Supplementary material for: Rib fractures in the elderly population: a systematic review
Source: Arch Orthop Trauma Surg. 2022 Feb 8;143(2):887–93. doi: 10.1007/s00402-022-04362-z (PMC9925562; doi:10.1007/s00402-022-04362-z)
Supplement: Supplementary file 2 — Supplementary file2 (DOCX 16 KB) [file 402_2022_4362_MOESM2_ESM.docx]

**Online resources Table 2.** Quality assessment according to the MINORS criteria.

| Criteria | Reported and adequate (2) | Reported but inadequate (1) | Not reported (0) |
| --- | --- | --- | --- |
| Clearly stated aim | Aim including outcomes reported | Aim reported without outcomes | Not reported |
| Inclusion consecutive patients | Inclusion/exclusion criteria reported | Unclear description inclusion/exclusion criteria | Not reported |
| Prospective collection data | Prospective | Not applicable | Not applicable |
| Appropriate endpoints | Appropriate endpoints to aim study | Endpoints not appropriate to aim study | Not reported |
| Unbiased assessment | Blinded evaluation of outcomes | Reason not blinding stated | Not reported |
| Appropriate follow-up | ≥ 1 year | < 1 year | Not reported |
| Loss to follow-up < 5% | ≤ 5% | > 5% | Not applicable |
| Prospective calculation study size | Prospective power-analysis performed | Prospective calculation without power-analysis | Not applicable |
| Adequate control group | ORIF versus MIPO treatment | Not applicable | Not applicable |
| Contemporary groups | Study/control group managed during same period | Study/control not managed during same period | Not reported |
| Baseline equivalence groups | Baseline characteristics described and comparable | Baseline characteristics not comparable | Not reported |
| Adequate statistical analyses | Statistical analysis described including type of analyses | Inadequate description statistical analysis | Not reported |

Items are scored 0 (not reported), 1 (reported but inadequate) or 2 (reported and adequate). The overall score ranging from 0 to 24 for comparative studies

# Rib fractures in the elderly population: A systematic review.

**Journal: Archives of Orthopaedic and Trauma Surgery**

Ruben J. Hoepelman^1,2^, Frank J.P. Beeres^2,3^, Marilyn Heng^4^, Matthias Knobe^2^, Björn-Christian Link^2^, Fabrizio Minervini^2^, Reto Babst^2,3^, Roderick. M. Houwert^1^, Bryan J.M. van de Wall ^2,3,^

1. Department of Trauma Surgery, University Medical Center Utrecht, Utrecht, the Netherlands

2. Department of Orthopedics and Trauma Surgery, Luzerner Kantonsspital, Lucerne, Switzerland

3. University of Lucerne, Department of Health Sciences and Medicine, Lucerne, Switzerland.

4. Department of Orthopedic Surgery, Harvard Medical School, Orthopedic Trauma Initiative, Massachusetts General Hospital, Boston, Massachusetts, USA

**Corresponding author:**

Bryan J.M. van de Wall, MD, PhD, E-mail address: Bryan.vandewall@luks.ch
