# Supplementary material for: Rib fractures in the elderly population: a systematic review
Source: Arch Orthop Trauma Surg. 2022 Feb 8;143(2):887–93. doi: 10.1007/s00402-022-04362-z (PMC9925562; doi:10.1007/s00402-022-04362-z)
Supplement: Supplementary file 3 — Supplementary file3 (DOCX 18 KB) [file 402_2022_4362_MOESM3_ESM.docx]

**Online resources Table 3.** Population, AIS scores and stratified ISS score per study

| Study | Fitzgerald^13^ | | Chen Zhu^12^ | | Pieracci^14^ | | Ali-Osman^16^ | | Kane^15^ | |
| --- | --- | --- | --- | --- | --- | --- | --- | --- | --- | --- |
| Population | 65+ and rib fractures | | 65+ and multiple rib fractures  Non chest AIS <3 | | 80+  Chest AIS ≥3  HeadAIS< 2 | | 60+ and ≥3 rib fractures | | 65+ and ≥3 rib fractures | |
|  | **NOM n(%)** | **RF n(%)** | **NOM n(%)** | **RF n(%)** | **NOM n(%)** | **RF n(%)** | **NOM n(%)** | **RF n(%)** | **NOM n(%)** | **RF n(%)** |
| Chest AIS  1  2  3  4  5 | NR  NR  NR  NR  NR | | 6(0.4)  32(2)  519(33.2)  161(10.3)  40(2.6) | 2(0.2)  33(3.2)  506(49.6)  193(18.9)  24(2.4) | NR  NR  NR  NR  NR | | NR  NR  NR  NR  NR | | NR  NR  NR  NR  NR | |
| Non-chest AIS  0  1  2 | NR  NR  NR | | 183 (11.7)  130 (8.3%)  445 (28.5) | 179(17.5)  146(14.3)  433(42.4) | NR  NR  NR | | NR  NR  NR | | NR  NR  NR | |
| Mean/median ISS  Stratified ISS  ISS <15  ISS 15-24  ISS >25 | 19 (14–23)  NRNR  NR | 21(16-26)  NR  NR  NR | NR  384 (24.6)  308 (19.7)  66 (4.2) | NR  369(36.1)  318(31.1)  71(7.0) | 13(4-34)  NR  NR  NR | 14 (4-57)  NR  NR  NR | 14 (8-24)  73(4.7)  30(2.9)  32(3.1) | 17.5 (9-25)  19(1.9)  24(2.4)  21(2.1) | 14.1 ±10.3  NR  NR  NR | 20.1±8.5  NR  NR  NR |

% are of all patients included in this review

NOM = nonoperative management

RF = rib fixation

AIS = abbreviated injury scale

ISS = Injury severity score

NR= Not reported

# Rib fractures in the elderly population: A systematic review.

**Journal: Archives of Orthopaedic and Trauma Surgery**

Ruben J. Hoepelman^1,2^, Frank J.P. Beeres^2,3^, Marilyn Heng^4^, Matthias Knobe^2^, Björn-Christian Link^2^, Fabrizio Minervini^2^, Reto Babst^2,3^, Roderick. M. Houwert^1^, Bryan J.M. van de Wall ^2,3,^

1. Department of Trauma Surgery, University Medical Center Utrecht, Utrecht, the Netherlands

2. Department of Orthopedics and Trauma Surgery, Luzerner Kantonsspital, Lucerne, Switzerland

3. University of Lucerne, Department of Health Sciences and Medicine, Lucerne, Switzerland.

4. Department of Orthopedic Surgery, Harvard Medical School, Orthopedic Trauma Initiative, Massachusetts General Hospital, Boston, Massachusetts, USA

**Corresponding author:**

Bryan J.M. van de Wall, MD, PhD, E-mail address: Bryan.vandewall@luks.ch
