# Supplementary material for: Rib fractures in the elderly population: a systematic review
Source: Arch Orthop Trauma Surg. 2022 Feb 8;143(2):887–93. doi: 10.1007/s00402-022-04362-z (PMC9925562; doi:10.1007/s00402-022-04362-z)
Supplement: Supplementary file 4 — Supplementary file4 (DOCX 17 KB) [file 402_2022_4362_MOESM4_ESM.docx]

**Online resources table 4.** Quality assessment of included studies.

|  | **Observationele studies** | Fitzgerald 2017 | Chen Zhu 2020 | Pieracci 2021 | Ali-Osman 2018 | Kane 2018 |
| --- | --- | --- | --- | --- | --- | --- |
|  |  |  |  |  |  |  |
|  |  |  |  |  |  |  |
| Clearly stated aim |  | 2 | 2 | 2 | 2 | 2 |
| Inclusion of consequetive patients |  | 2 | 2 | 2 | 2 | 2 |
| Prospective data collection |  | 0 | 0 | 0 | 0 | 0 |
| Appropriate endpoints |  | 2 | 2 | 2 | 2 | 2 |
| Unbiased assessment endpoints |  | 0 | 0 | 0 | 0 | 0 |
| Appropriate follow-up (>1year) |  | 1 | 0 | 0 | 0 | 0 |
| Loss-to-follow-up <5% |  | 1 | 0 | 0 | 0 | 0 |
| Prospective calculation study size |  | 0 | 0 | 0 | 0 | 0 |
| Adequate control group |  | 2 | 2 | 2 | 2 | 2 |
| Contemporary groups |  | 1 | 2 | 2 | 1 | 1 |
| Baseline quivalence of groups |  | 2 | 2 | 1 | 1 | 1 |
| Adequate statistical analysis |  | 2 | 2 | 2 | 2 | 2 |
| Total score |  | 15 | 14 | 13 | 12 | 12 |

# Rib fractures in the elderly population: A systematic review.

**Journal: Archives of Orthopaedic and Trauma Surgery**

Ruben J. Hoepelman^1,2^, Frank J.P. Beeres^2,3^, Marilyn Heng^4^, Matthias Knobe^2^, Björn-Christian Link^2^, Fabrizio Minervini^2^, Reto Babst^2,3^, Roderick. M. Houwert^1^, Bryan J.M. van de Wall ^2,3,^

1. Department of Trauma Surgery, University Medical Center Utrecht, Utrecht, the Netherlands

2. Department of Orthopedics and Trauma Surgery, Luzerner Kantonsspital, Lucerne, Switzerland

3. University of Lucerne, Department of Health Sciences and Medicine, Lucerne, Switzerland.

4. Department of Orthopedic Surgery, Harvard Medical School, Orthopedic Trauma Initiative, Massachusetts General Hospital, Boston, Massachusetts, USA

**Corresponding author:**

Bryan J.M. van de Wall, MD, PhD, E-mail address: Bryan.vandewall@luks.ch
